# Use of Balint Groups (BGs) to Support Wellbeing Amongst Medical Students in a Public Health Institution

**DOI:** 10.1192/bjo.2025.10312

**Published:** 2025-06-20

**Authors:** Lay Ling Tan, Karen Koh

**Affiliations:** Changi General Hospital, Singapore, Singapore

## Abstract

**Aims:** BG is named after its founder, Michael Balint, a trained therapist. BGs are reflective practice groups and have been shown to help with critical reflection, self-discovery and deep connection to others. It can enhance empathic understanding of the doctor-patient therapeutic relationship and has been used to facilitate reflections amongst doctors and other healthcare professionals. Its application in medical students have not been widely studied.

Loss of empathy and burnout as they began clinical training have been widely reported amongst medical students. As part of our hospital’s initiative to support their wellbeing, our Education Office offered medical students the experience of BGs by trained facilitators. We aim to understand students’ experiences of attending BGs and its impact on their wellbeing.

**Methods:** Students were invited via an email detailing the purpose and structure of BG. A non-random purposive sample of medical students with clinical postings of at least 6 weeks were included. BGs were conducted based on the students’ clinical groupings. Data was collected through self-reported questionnaires and qualitative feedback to capture the students’ experience with BGs in the clinical setting.

**Results:** 58 medical students participated in 6 sessions of BG conducted in groups of 8 to 10 with 1 or 2 facilitators. About 70% of the students were better able to appreciate the psychological aspects of patient encounters and endorsed that they benefited from facilitation of reflections during BGs. Content analysis of qualitative feedback supported the following themes: 1. Safe space to reflect on difficult clinical encounters; 2. Improved self-awareness; 3. Appreciation of different perspectives.

**Conclusion:** Providing the students with a safe therapeutic space to share their thoughts about difficult clinical encounters supported critical reflection and develop others’ awareness as they consider different perspectives. The sense of camaraderie and togetherness provided by the group may help to build empathy amongst our students and address burnout and compassion fatigue. BGs can also foster a sense of belonging and support during medical students’ stressful clinical years with the provision of a safe and secure space to explore emotions and attitudes. There are ongoing efforts to incorporate such reflective groups systematically into the undergraduate medical education clinical curriculum.

